# Further evidence regarding the effect of KAMs on audit report lag

**DOI:** 10.1371/journal.pone.0320183

**Published:** 2025-03-25

**Authors:** Ayşegül Ciğer, Bülent Kınay, Murat Ocak

**Affiliations:** 1 Department of Finance and Banking, Faculty of Applied Sciences, Akdeniz University, Antalya, Türkiye; 2 Department of Accounting and Tax Applications, Social Sciences Vocational School, Akdeniz University, Antalya, Türkiye; 3 Management Information Systems Department, Uzunkopru School of Applied Sciences, Trakya University, Edirne, Türkiye; Shanghai Business School, CHINA

## Abstract

This paper investigates the effect of the number of key audit matter disclosures (KAMs) on audit report lag, focusing on Turkey, an emerging country. The main findings indicate that the number of KAMs positively influences audit report lag in Turkey. System GMM results reinforce our primary estimations, supporting the robustness of our findings. Notably, auditing by large audit firms moderates the effect of KAM numbers of on audit report lag. We categorized KAMs into four sub-types and found that only revenue-related KAMs significantly increase audit report lag. Additionally, various corporate governance, audit firm, and individual auditor attributes influence the number of KAMs reported.

## 1. Introduction

The International Auditing and Assurance Standards Board (IAASB) has approved the standard ISA 701 to disclose key audit matters (KAMs hereafter) in audit reports to make them more informative and enhance their communication value for third parties [1]. Individual auditors are required to disclose companies’ significant risks as KAMs in their auditor reports. Thus, KAMs play an important role in improving the scope, originality, and transparency of auditor reports. The inclusion of KAMs in auditor reports has become a compelling area of accounting research, with many studies examining whether KAMs are informative [2,3], if they increase audit quality [4,5], factors influencing KAMs, and issues related to KAMs [6].

According to the Conceptual Framework for Financial Statements issued by IASB, timeliness is a crucial characteristic of financial statements [7]. Financial information should be presented promptly to influence third party decisions. KAM requirements are a double-edged sword: while KAMs enhance the communication value of audit reports [2], and play an essential role in investor decision-making [3], they may also require auditors to exert more effort and spend additional time preparing and presenting KAMs in audit reports [8–10].

Although several studies demonstrate a positive association between the number of KAMs (or their features) and audit reporting lag [11,12], there remains a gap in understanding this relationship within emerging countries, such as Turkey. While the number of KAMs typically increases audit report lag, large audit firms may mitigate this association due to their resources, skilled staff, and technological advantages [13–15]. Additionally, certain types of KAMs may have a greater impact on audit report lag than others, as some items are more complex to audit [16]. The need for comprehensive analysis of different KAM types and their effects on audit report lag in emerging markets underscores the importance of examining how audit firm size and specific KAM types influence the audit reporting process in these contexts.

This paper addresses several research questions. First, does the number of KAMs in the audit reports positively affect audit report lag? Second, does audit firm size moderate the effect of the number of KAMs on audit report lag? Third, which types of KAM disclosures most significantly affect audit report lag? Finally, what factors drive the number of KAMs?

Our study employs companies quoted in Borsa Istanbul, along with their audit firms and individual auditors, to investigate these questions. The sample comprises 602 observations between 2017 and 2021 in Turkey. Using a Turkish sample is appropriate since Turkey is an emerging market, and audit research generally focuses on companies or audit firms in large and developed countries. As in many developing nations, data availability is limited in Turkey.

Except for financial data, all data were collected manually. For example, corporate governance variables, such as board independence and gender diversity, were manually sourced from companies’ annual reports, while KAM disclosures were manually retrieved from audit reports. These findings, which control for a set of variables related to audit firms, individual auditors, and corporate governance, may provide valuable insights into the unique characteristics of Turkish audit firms, auditors, and company structures. Financial data about companies are obtained from Finnet database.

The rationale behind using the Turkish sample is as follows. First, Turkey adheres to international auditing standards, including the KAM reporting standard [1]. In Turkey, many auditors at audit firms are former tax auditors with experience in government tax institutions, which may lead to differences in the variety of KAMs. For instance, former tax auditors may focus more on revenue-related items in the income statement that could impact taxes, potentially causing delays in the audit process [16].

Second, the ownership structures of publicly listed Turkish companies are characterized by high levels of concentration, as one or more majority shareholders (i.e., families) hold significant blocks of shares in the company [17,18]. Board members, particularly chairpersons, generally have a large share in the ownership structure of companies. KAMs are chosen from the topics discussed with those responsible for company governance [1,19]. Boards of directors, as the top governance mechanism in companies, may not wish risky issues to be disclosed as KAMs because several board members, who own significant shares in the company, may find such disclosures contrary to their interests [19]. On the other hand, audit firms or individual auditors concerned about their reputation may request that these risky issues be disclosed as KAMs. This situation could turn into a negotiation between the parties, potentially increasing the audit report lag of companies.

Third, Turkey is characterized by a high level of power distance, low uncertainty tolerance, and a collectivist social structure [20,21]. Power distance influences communication and decision-making frameworks. In cultures with high power distance, there is a strong preference for centralized authority, top-down communication, and restricted information sharing. Such cultures support environments that maintain hierarchical structures and limit the flow of information [22]. While KAMs may enhance the communication value of audit reports [2], in a country like Turkey, which is characterized by power distance, this trait might influence corporate reporting policies and contribute to delays in audit reports. For instance, slow communication of information between the audit firm and the company regarding KAMs could be a significant factor affecting the timeliness of disclosures in certain contexts [23].

Fourth, Turkey faces economic instability, which also affects companies. Operating in such an environment influences the reported KAMs and the decisions of auditors and management, thereby impacting reporting timeliness [4]. Comparing these results with those from other countries, particularly through moderating analyses and examining the types of KAMs that contribute to reporting delays, will add to the literature.

We investigate the impact of the number of KAMs on audit report lag and examine how audit firm size moderates this relationship. Additionally, we explore which types of KAMs influence audit report lag. Our primary analytical methods include Ordinary Least Squares (OLS) and Poisson regressions, and we further validate our findings using the System Generalized Method of Moments (GMM). Lastly, we analyze the determinants of KAMs using OLS and Poisson estimation procedures.

In this paper, we explore the potential impact of KAMs on audit report lag in Turkey. First, we confirm findings from Bepari et al. [24], which indicate an increase in audit report lag following the introduction of KAM reporting in the Bangladesh. We find that the number of KAMs positively affects audit report lag, and our main results remain robust even when addressing endogeneity issues with the system GMM. Furthermore, a unique finding of our study is that large audit firms can mitigate the effect of the number of KAMs on audit report lag due to their resources and staff capabilities. We categorize KAMs into four sub-categories: asset-related KAMs, liability-related KAMs, revenue-related KAMs, and other KAMs. Notably, revenue-related KAMs have the most substantial impact on audit report lag. Lastly, we assess the determinants of KAMs by examining corporate governance levels, individual auditor attributes, and audit firm characteristics specific to Turkish firms.

This paper contributes to the KAM literature in several ways. Recent studies show mixed findings on the effect of KAMs on audit report lag across different countries [8,24]. Bepari et al. [24] found an increase in audit report lag post-KAM reporting. This paper provides evidence of a positive effect of the *number* of KAMs on audit report lag in Turkey, an emerging economy. Validating recent findings on KAM-related delays using hand-collected data from Turkey is meaningful. This study offers insights into a developing market, expanding research beyond Western-Centric contexts. Individual auditors are responsible for preparing audit reports and their professional judgement may be influenced by their background and the country where they conduct their work. Although recent studies use audit firm size as a control variable, few examine whether audit firm size affects the relationship between KAMs and audit report lag [11,12] This study provides initial evidence of audit firm size’s moderating effect on this relationship. While KAMs can delay report publication, this effect is mitigated when larger audit firms conduct audits, given their extensive resources and sizable staff. Third, we categorize KAMs into four groups: asset-related KAMs, liability-related KAMs, revenue-related KAMs, and other KAMs, identifying which sub-categories most significantly impact on audit report delays. This issue has not been addressed in recent research. Our findings suggest that revenue-related KAMs contribute most to audit report lag, which may reflect the tax-oriented background of some Turkish auditors. Additionally, we examine the determinants of KAMs by analyzing company, individual auditor, audit firm, and corporate governance factors within Turkish companies. This study also emphasizes that auditor attributes, such as education level and international experience, influence the number of KAMs.

Based on our findings, we suggest several managerial policy implications. Companies and audit firms should allocate adequate time and resources early in the audit cycle to address complex audit areas, as more KAMs can extend audit report lag. Engaging larger audit firms may ensure timely reporting for audits with numerous or complex KAMs. Prioritizing KAMs that impact audit report lag can improve internal controls and documentation. Investing in advanced audit technology and ongoing training for internal audit teams on emerging risks can streamline the process and reduce delays. Strengthening collaboration between management and auditors through regular communication and early identification of potential KAMs can lead to a more efficient audit process. Regularly updating internal policies based on common KAMs and audit findings can mitigate issues that contribute to audit report lag, thereby improving audit efficiency and financial reporting timeliness.

Section 2 provides an overview of Turkey’s audit environment. Section 3 reviews relevant literature review and develops the study’s hypotheses. Section 4 describes the research design, including sample selection, estimation models, and variables. Section 5 presents the study’s results, and Section 6 concludes with implications.

## 2. Institutional settings

Turkey has adopted International Auditing Standard 701 [1], mandating KAM reporting for publicly traded companies on the Borsa Istanbul stock exchange as of January 1, 2017. Borsa Istanbul began operations in 1986 with 19 companies; as of 2022, it hosts 444 companies, the majority of which are manufacturing firms [25]. Financial statements of companies listed on Borsa Istanbul are required to be audited by an independent audit firm. As Fan [26] noted, monitoring individual auditors and audit firms serves not only as a tool for sanctioning but also as a means to identify issues and offer recommendations. In Turkey, the Public Oversight Authority (POA) oversees independent audit firms and individual auditors.

Independent audit firms began operating in Turkey during the 1960s and 1970s, prompted by the demand for independent audits from foreign investors [27]. Since 2011, the Public Oversight Authority Board (POAB) has been authorized to regulate audits, audit firms, and individual auditors. According to POA statistics [28], 17,589 individual auditors are registered, and 389 audit firms are authorized by the POA. However, most of these audit firms and individual auditors do not audit the financial statements of companies listed on Borsa Istanbul. According to statistics from Turkey’s Public Disclose Platform, only 94 audit firms and their individual auditors audit the financial statements of Borsa Istanbul-listed companies.

## 3. Literature review and hypothesis development

### 3.1. Literature review

Manoel and Quel [29] suggest that KAMs positively impact the reduction of the audit expectations gap, as they provide detailed information on sensitive matters identified by auditors during the audit process. Sirois et al. [2] note that third parties pay particular attention to KAM-related disclosures audit reports, enhancing the communication value of these reports. Zhai et al. [30] provide causal evidence that KAMs reduce stock price synchronicity by delivering increased company-specific information. Liao et al. [31] find that KAMs are not significantly associated with the risk of stock price collapse and do not influence information transparency or managerial opportunistic behavior. Kong et al. [32] report that KAMs provide useful insights and impact analysts’ knowledge acquisition. Köhler et al. [33], Moroney et al. [34] and Ong et al. [3] indicate that KAM disclosures are influential in investors decisions, although non-professional investors may not utilize KAM information in their investment decisions [33]. In contrast, Ma et al. [35] explore the association between KAMs and investors’ decision making and find that KAMs disclosed by small audit firms can mislead non-professional investors. Moroney et al. [34] also state that investors perceive the audit as less valuable and less credible when non-Big Four audit firms include KAMs in audit reports.

Ong et al. [3] emphasize that KAMs are essential for third parties but need to be readable to be useful to investors. Some researchers argue that more readable KAM disclosures reduce information asymmetry and positively affect credit ratings and interest rates, as they lower information asymmetry and creditors’ risk perception [36–39]. Huang et al. [40] find that firms with less readable, negatively toned, and highly detailed KAM disclosures are more likely to engage in financial restatements. Sneller et al. [41] report that technology-related KAM disclosures attract increased attention from investors. Daugherty et al. [42] and Elmarzouky et al. [43] note that the KAM process raises audit fees, while Gold et al. [4] and Espahbodi et al. [5] find that KAMs improve financial report quality. The rationale for increased audit fees and financial reporting quality is that individual auditors put additional effort into their work and to comply with ISA 701’s KAM requirements. Kitiwong et al. [44] and Lin and Yen [45] argue that changes in auditors, where the incoming auditor identifies different KAMs related to the company’s significant risks, affect the quality of financial reporting due to alterations in the audit plan and process. On the other hand, Fera et al. [46] report that companies with strong governance systems tend to have fewer KAMs, as effective governance can ease tensions between the audit firm and the company. An interesting study by Rousseau and Zehms [47] reveals that audit partner decision styles influence KAM outcomes more than audit firm styles. Companies audited by the same partner receive KAMs that are 10% more textually similar compared to those audited by different partners.

### 3.2. Hypothesis development

According to agency theory, corporate risk disclosure helps reduce information asymmetry between managers and investors [48,49]. The value of accounting information for financial statement users depends on its completeness, accuracy, reliability, and timeliness [50]. Schwartz and Soo [51] suggest that reporting disputes may cause delays, while some clients prioritize traditional reporting practices or seek improved timeliness. Timely financial reports enhance the value of accounting information. From an agency theory perspective, reporting KAMs can reduce information asymmetry between companies and third parties. However, reporting additional KAMs may delay audit reports, creating an agency cost due to the time-intensive nature of the process.

Several studies have examined the impact of the number of KAMs on audit report lag across different countries, with mixed findings [8–10,52,53]. Abdullatif et al. [8] argue that individual auditors exert greater effort on procedures related to KAM items, which increases audit report lag. However, they found no significant association between audit report lag and KAMs in the Jordan. Bepari et al. [52] observed that audit report lag increased in the post-KAM reporting period in Bangladesh, attributing this to auditors’ increased efforts to disclose KAM-specific reasons. Batwaah et al. [9] and Bédard et al. [10] also suggest that individual auditors may spend more time preparing and reviewing audit reports to fulfill KAMs requirements, thus increasing audit report lag. However, Batwaah et al. [9] found that KAMs did not increase audit report lag as expected, attributing this to audit firms’ allocation of experienced auditors to mitigate delays. Similarly, Bédard et al. [10] found no association between first-time KAM implementation or new KAM reporting and audit report lag.

Küster [11] explored determinants of KAM linguistic features, such as readability and word count, finding that audit report lag negatively and significantly affects KAM readability. Preparing more readable KAMs can delay audit reports. Federsel and Hörner [12], using a sample from European countries, found that the number of KAMs positively and significantly affects audit report lag, though the relationship varies by country.

We propose that individual auditors or audit firms exert additional effort [8] and dedicate more time to meet KAM requirements, particularly in addressing procedures related to KAM items [11,12,52]. Therefore, as more KAMs are reported, audit report lag is likely to increase. Based on prior findings on the potential positive effect of KAMs on audit report lag, we hypothesize the following relationship in the Turkish context:

H1. There is a positive and significant association between the number of KAMs and audit report lag.

As noted, reporting KAMs can reduce information asymmetry between companies and third parties, but may delay audit reports. However, larger audit firms may complete audits more quickly than smaller firms [46], owing to their greater experience, competence, and larger pool of staff. Large audit firms help reduce agency costs, such as reporting delays, due to their greater resources.

Large audit firms, with their experienced staff and access to advanced technologies, can perform audits more efficiently with greater flexibility than smaller firms [13,49]. They are better able to attract skilled personnel, invest in training their staff, and implement new audit technologies [14]. Large audit firms also have a more refined risk-based audit approach and can quickly assess governance risks [55]. Technology significantly impacts the audit process, enabling software to analyze control risks quickly [15]. Large audit firms invest heavily in technology to streamline audits [56]. Thus, while individual auditors may exert more effort and time to meet KAM requirements [8,9], large audit firms, with skilled staff and advanced technology, can counteract the delays caused by KAMs disclosures [13,14,49]. Additionally, they can also reverse the reporting lag caused by the KAMs by using their technology effectively [15,56]. Larger audit firms often allocate ample time and resources early in the audit cycle, leveraging their human resources and technological investments to handle KAMs more efficiently. We propose the following hypothesis:

H2. Audit firm size moderates the effect of the number of KAMs on audit report lag.

Recent studies analyzing the types of KAMs reported in audit reports reveal that revenue-related KAMs are the most commonly reported [57,58]. Revenue recognition is a central issue in financial scandals, with primary audit deficiencies often involving revenue and accounting estimates [59]. Companies are more likely to manipulate revenue and assets [60], and individual auditors spend considerable time auditing these items [16]. Our content analysis of the sample reveals that revenue-related KAMs are the most frequently reported. Since revenue recognition is complex and high-risk, individual auditors expend more effort to report KAMs related to revenue compared to other accounts. Thus, our final hypothesis is as follows:

H3. The number of revenue-related KAMs has a greater effect on audit report lag than the number of asset-related, liabilities-related, and other KAMs.

## 4. Research design

### 4.1. Sample selection

We selected companies, audit firms, and individual auditors in Turkey to test our hypotheses. In Turkey, KAMs have been disclosed in the audit reports since 2017. Our sample spans from 2017 to 2022, as data for 2023 has not yet been published. Financial, insurance, investment fund, and mining companies are excluded due to differing reporting procedures. Our sample includes 602 observations and six years (2017-2022), as presented in Table 1.

**Table 1:** Sample.

Industry/Year	2017	2018	2019	2020	2021	2022	Total
Information and Communication	5	5	4	2	1	1	18
Electricity Gas and Water	4	3	3	4	4	4	22
Hotels and Restaurants	5	5	5	3	2	2	22
Technology	9	9	9	8	9	9	53
Wholesale and Retail Trade	12	11	13	7	6	6	55
Transportation	3	3	3	1	1	1	12
Professional Activities	2	2	2	2	2	1	11
Manufacturing	86	87	89	56	43	40	401
Construction and Public Works	0	0	0	4	2	2	8
Total	126	125	128	87	70	66	602

Information regarding KAMs are manually collected from audit reports. Individual auditor attributes, such as gender and education level including auditors’ names, were gathered from audit firm transparency reports and LinkedIn profiles. Audit firm size data was obtained from transparency reports, and company-specific characteristics, such as size and leverage, were sourced from the Finnet Database [61]. Finnet is a data provider regarding the financial structures of companies quoted in Borsa Istanbul. Data on companies’ assets, liabilities and income statement structures are available. Additionally, basic ratios can be accessed from this database. Corporate governance data at the company level was manually collected from annual reports.

### 4.2. Estimation models

The first model estimates the effect of the number of KAMs on audit report lag (H1). The model is as follows:

LnLag (Lag or AdjLag) =β_0_ +  β_1_ LnTotalKAM (or AdjKAM) +  β_2_ AuditorEdu +  β_3_ AuditorGender +β_4_ AuditorAbroad +  β_5_ LnAuditorTenure +  β_6_ LnAFSize +  β_7_ Duality +  β_8_ BoardInd +  β_9_ BoardFemale +  β_10_ LnBoardSize +  β_11_ Leverage +  β_12_ Loss +  β_13_ MTB +  β_14_ LnSize +  β_15_ Growth +  β_16_ LnAge +  Industry Fixed +  Year Fixed +  error term(1)

We expect that a higher number of KAMs will positively affect audit report lag. Audit reports with more KAMs tend to experience delays. We use three dependent variables: the logarithmic value of audit report lag (*LnLag*), the raw value of audit report lag (*Lag*), and industry-year adjusted reporting lag (*AdjLag*). We run Ordinary least square and Poisson regressions using the natural logarithmic (OLS) and raw values (Poisson) of the audit report lag, respectively. We use OLS when employing the industry-year adjusted lag variable.

The second model tests the interaction effect between the number of KAMs and audit firm size on audit reporting lag (H2). We create an interaction variable (LnTotalKAM*LnAFSize and AdjKAM*LnAFSize) using the number of KAMs (*LnTotalKAM* and *AdjKAM*) and audit firm size (*LnAFSize*) and expect this to negatively affect audit report lag. The second model is as follows:

LnLag (Lag or AdjLag) =β_0_ +  β_1_ LnTotalKAM (or AdjKAM) +  β_2_ LnTotalKAM *  LnAFSize (or AdjKAM*LnAFSize) +  control variables +  Industry Fixed +  Year Fixed +  error term (2)

The third model tests the effect of different types of KAMs on the audit report lag (H3). We divide KAMs into four categories: asset-related KAMs (*LnTotalKAMType*_*Assets*_*, AdjKAMType*_*Assets*_), liability-related KAMs (*LnTotalKAMType*_*Liabilities*_*, AdjKAMType*_*Liabilities*_), revenue-related KAMs (*LnTotalKAMType*_*Revenues*_*, AdjKAMType*_*Revenues*_), and other-related KAMs (*LnTotalKAMType*_*Others*_*, AdjKAMType*_*Others*_). The model is as follows:

LnLag (Lag or AdjLag) =β_0_ +  β_1_ LnTotalKAMType_Assets, Liabilities, Revenues, Others_ (or AdjKAMType_Assets, Liabilities, Revenues, Others_) +  control variables +  Industry Fixed +  Year Fixed +  error term(3)

#### 4.2.1. Variables used to test H1.

To test H1, we employ different versions of the audit report lag as the dependent variable in our models. For the OLS estimation, we use the natural logarithmic value of the total number of the days following the fiscal year-end until the audit report date (*LnLag*) as the dependent variable [10]. In the Poisson regression, the raw count of the total days between the fiscal year-end and the audit report date (*Lag*) is used as the dependent variable [8]. We also include industry-year adjusted reporting lag (*AdjLag*), which measures a company’s audit report lag relative to the industry-year mean.

Recent studies by Rahaman and Karim [49], Hegazy et al. [62], Pinto and Morais [6] use the number of KAMs as a dependent or test variable to analyze factors influencing KAM disclosure, such as auditor characteristics. We apply the natural logarithmic value of total number of KAMs (*LnTotalKAM*) as the variable of interest. Additionally, we employ industry-year adjusted KAMs (*AdjKAM*) following Rousseau and Zehms [53], which is calculated as total number of KAMs in a company’s audit report minus the industry-year mean. *LnTotalKAM* and *AdjKAM* are the primary variables to test H1.

Control variables are applied at four levels. The first two levels control for individual auditor and audit firm characteristics, including individual auditor gender (*AuditorGender*), individual auditor education level (*AuditorEdu*), individual auditor tenure (*AuditorLnTenure*), individual auditor foreign experience (*AuditorAbroad*), and audit firm size (*LnAFSize*). Female auditors (*AuditorGender*) tend to take longer to complete audits [63], while auditors with a master’s or Ph.D. (*AuditorEdu*) may spend less time on audits due to their advanced competency [64]. We control for auditor tenure (*LnAuditorTenure*) because a longer tenure can lead to more efficient audits as the auditor becomes familiar with the client’s operations, financial statements, and internal control systems [65–67]. Individual auditors with foreign experience (*AuditorAbroad*) may require additional time due to unfamiliarity with local contexts [68]. We also control for audit firm size (*LnAFSize*), as large audit firms have greater resources and staff, enabling more efficient audits [13,54].

Third, we control for company characteristics, including size (*LnSize*), leverage (*Leverage*), performance (*Loss*), age (*LnAge*), growth (*Growth*), and market-to-book value (*MTB*). Larger (*LnSize*) and older companies (*LnAge*) may have stronger internal systems; reducing the time required for audits [69]. High-leverage companies *(Leverage)* and those reporting losses (*Loss*) generally require more auditing time due to the complexity of verifying their financials as they tend to declare bad news later [70,71].

Finally, we control for corporate governance factors: board gender diversity (*BoardFemale*), board independence (*BoardInd*), board size (*LnBoardSize*), and duality (*Duality*). Female board members (*BoardFemale*) tend to improve risk monitoring, possibly increasing reporting lag [72,73]. Independent directors (*BoardInd*) enhance monitoring [74], while larger boards (*LnBoardSize*) are generally more efficient in executing their responsibilities, potentially reducing lag [46]. Dual roles of the chairperson and CEO (*Duality*) may suggest financial irregularities in their financial statements, extending audit time [75]. We fix years and industries in the estimation models. All variable measurements are in S1 Table.

#### 4.2.2. Variables used to test H2.

Large audit firms have skilled and experienced staff [13,14,54] and advanced technology to perform their audits [15,56]. To test the moderating effect of audit firm size on audit report lag (H2), we create interaction variables combining audit firm size (*LnAFSize*) with the total number of KAMs (*LnTotalKAM* and *AdjKAM*). The interaction terms (LnTotalKAM*LnAFSize and AdjKAM*LnAFSize) test H2. The other control and dependent variables remain consistent with those mentioned in Section 4.2.1, as detailed in S1 Table.

#### 4.2.3. Variables used to test H3.

Following the studies by Filipović et al. [57] and Pérez et al. [58], we examine whether revenue-related KAMs have a distinct effect on audit report lag, as revenues often require more audit time [16]. We categorize KAMs into four subgroups to analyze their impact on audit report lag: assets-related KAMs (*LnTotalKAMType*_*Assets*_, *AdjKAMType*_*Assets*_), liability-related KAMs (*LnTotalKAMType*_*Liabilities*,_ *AdjKAMType*_*Liabilities*_), revenue-related KAMs (*LnTotalKAMType*_*Revenues*,_ *AdjKAMType*_*Revenues*_) and other-related KAMs (*LnTotalKAMType*_*Others*_, *AdjKAMType*_*others*_). The headings in Table 2 are grouped into these four sub-components, based on the balance sheet and income statement. These subgroups test H3. All control variables and dependent variables are mentioned in Section 4.2.1, with measurements shown in S1 Table.

**Table 2:** Descriptive statistics of the sample.

Panel A: Descriptive Statistics						
Variable	Obs	Mean	Median	Std. Dev.	Min	Max
Lag	602	59.52	60	11.09	29	131
LnLag	602	4.068	4.0943	0.193	3.367	4.875
AdjLag	602	0.007	0.034	0.185	-0.670	0.814
TotalKAM	602	2.143	2	1.244	1	7
LnTotalKAM	602	0.611	0.693	0.543	0	1.946
AdjKAM	602	0.631	0.482	1.200	-2.666	5.566
AuditorEdu	602	0.191	0	0.393	0	1
AuditorGender	602	0.106	0	0.308	0	1
AuditorAbroad	602	0.219	0	0.414	0	1
AuditorTenure	602	2.226	2	1.291	1	7
LnAuditorTenure	602	0.638	0.693	0.570	0	1.946
AFSize	602	253.0	121	244.0	5	699
LnAFSize	602	4.660	4.795	1.552	1.609	6.550
Duality	602	0.174	0	0.380	0	1
BoardInd	602	0.325	0.333	0.069	0.142	0.5
BoardFemale	602	0.153	0.142	0.149	0	0.600
BoardSize	602	7.217	7	2.188	3	15
LnBoardSize	602	1.935	1.945	0.278	1.098	2.708
Leverage	602	0.545	0.581	0.233	0.042	0.998
Loss	602	0.211	0	0.408	0	1
MTB	602	0.049	0.020	0.249	0.003	5.372
LnSize	602	20.57	20.34	1.889	16.43	25.80
Growth	602	0.332	0.242	0.363	-0.460	2.422
Age	602	37.54	37	14.80	7	78
LnAge	602	3.529	3.610	0.472	1.946	4.357
**Panel B: Frequency table of binary variables**						
Variable		0	1	0 (%)	1 (%)	
AuditorGender	602	538	64	89.37	10.63	
AuditorEdu	602	487	115	80.90	19.10	
AuditorAbroad	602	470	132	78.07	21.93	
Duality	602	497	105	82.56	17.44	
Loss	602	475	127	78.90	21.10	
**Panel C: Tabulation of KAMs**						
Number of KAMs	Freq.	Percent		Cum		
1	226	37.54		37.54		
2	192	31.89		69.44		
3	106	17.61		87.04		
4	46	7.64		94.68		
5	20	3.32		98.01		
6	6	1.00		99.00		
7	6	1.00		100.00		
Total	602	100.00				
**Panel D: Types of KAMs**						
**Type**		**Number**		**Type**		**Number**
Inventories		128		Trade payables		15
Provisions		37		Financial payables		50
Cash and equivalent assets		9		Financial investments		22
Receivables		213		Fair value		15
Revenues		314		Contract of concession		5
Tangible assets		200		Right of usufruct		23
Investment properties		82		Derivatives		7
Intangible assets		90		Current period tax		1
Income taxes		41		Others		21
Related parties		21				

## 5. Results

### 5.1. Descriptive statistics and correlation matrix

Panel A of Table 2 provides descriptive statistics. The mean audit report lag (*Lag*) is approximately 60 days, ranging from 29 days to 131 days, with a mean *LnLag* is 4.068. The number of KAMs averages around 2, with a minimum of 1 and a maximum of 7. The average difference between a company’ audit lag and the industry-year mean audit report lag is 0.007. The mean difference between the number of KAMs of a company and the industry average is 0.631, with a minimum of -2.666 and a maximum of 5.566. On average, companies have about one more KAM than the industry average. While one company reported approximately 3 fewer KAMs than its industry, another company reported approximately 6 fewer KAMs than its sector.

Comparing the mean audit report lag (*Lag)* in Turkey with studies on Jordan by Abdullatif et al. [8] and Oman by Baatwah et al. [9], our sample’s mean audit report lag is higher than Baatwah et al.’s findings [9] but lower than those reported by Abdullatif et al. [8].

The average auditor tenure with a company (*AuditorTenure*) is about 2 years with audit firms averaging 253 employees (*LnAFSize*). The board independence percentage (*BoardInd*) and female representation (*BoardFemale*) averages are 32.5% and 15.3%, respectively. The mean board size (*BoardSize*) is approximately 7 members. Mean values for *Leverage*, *MTB*, *Growth*, *LnSize,* and *FirmAge* are 0.545, 0.049, 0.332, 20.57, and 37.54, respectively.

Panel B of Table 2 shows the binary variable frequencies. Female auditors (*AuditorGender*) represent 10.63% of the sample, with 19.10% holding a master’s or Ph.D. (*AuditorEdu*) and 21.93% having international experience (*Abroad*). CEOs also serving as chairpersons (*Duality*) are found in 17.44% of cases, while 17.44% report losses (*Loss)*.

Panel C of Table 2 indicates that in 266 observations, only one KAM is disclosed, while some cases disclose up to six KAMs. Panel D of Table 2 lists the types of KAMs disclosed, with revenues as the most frequently reported item.

The correlation matrix in Table 3 addresses potential multicollinearity issues. There is no multicollinearity among control variables, with the highest coefficient (0.625) between *LnBoardSize* and *BoardInd* [76]. High correlations are noted between *Lag*, *LnLag*, and *AdjLag*, as well as among the primary variables of interest. However, as each is run in a separate model, this is not problematic. Additionally, we expect a positive and significant association between the variables of interest and the dependent variables. The associations between the variables of interest and the dependent variables are positive but generally insignificant. It should be noted that the correlation matrix was created solely to detect multicollinearity, not for hypotheses testing.

**Table 3:** Correlation matrix.

	1	2	3	4	5	6	7	8	9	10	11	12	13	14	15	16	17	18	19	20
LnLag (1)	1																			
Lag (2)	0.981***	1																		
AdjLag (3)	0.967***	0.951***	1																	
LnTotalKAM (4)	0.043	0.049	0.076*	1																
AdjKAM (5)	0.053	0.058	0.082**	0.941***	1															
AuditorEdu (6)	-0.022	-0.034	-0.037	0.143***	0.177***	1														
AuditorGender (7)	-0.073*	-0.049	-0.071*	0.074*	0.070*	-0.072*	1													
AuditorAbroad (8)	-0.013	-0.001	-0.024	-0.147***	-0.138***	-0.040	0.050	1												
LnAuditorTenure (9)	0.001	0.005	0.001	-0.029	-0.023	0.002	0.020	0.007	1											
LnAFSize (10)	-0.172***	-0.154***	-0.181***	-0.218***	-0.252***	-0.186***	0.048	0.393***	0.038	1										
Duality (11)	0.098**	0.090**	0.115***	-0.052	-0.041	0.031	-0.066	-0.114***	0.023	-0.250***	1									
BoardInd (12)	0.082**	0.098**	0.101**	0.030	0.046	-0.036	-0.081**	-0.148***	-0.040	-0.283***	0.037	1								
BoardFemale (13)	0.106**	0.095**	0.105**	0.002	-0.003	0.047	-0.062	-0.113***	-0.027	-0.074*	0.078*	0.048	1							
LnBoardSize (14)	-0.213***	-0.208***	-0.233***	-0.059	-0.089**	-0.022	0.076*	0.273***	0.095**	0.376***	-0.117***	-0.625***	-0.148***	1						
Leverage (15)	0.020	0.030	-0.027	0.011	-0.006	0.063	0.109***	0.112***	0.006	0.235***	0.014	-0.186***	-0.062	0.199***	1					
Loss (16)	0.177***	0.171***	0.196***	0.014	0.008	-0.058	0.088**	-0.083**	0.096**	-0.017	0.043	-0.058	-0.074*	-0.020	0.212***	1				
MTB (17)	-0.055	-0.057	-0.070*	0.047	0.051	0.110***	0.028	-0.025	0.016	0.063	-0.047	-0.094**	-0.037	0.130***	0.152***	0.114***	1			
LnSize (18)	-0.091**	-0.082**	-0.136***	0.066	0.024	-0.025	0.078*	0.293***	0.085**	0.447***	-0.034	-0.194***	-0.082**	0.503***	0.357***	-0.165***	0.058	1		
Growth (19)	0.013	0.015	-0.019	0.067	0.048	0.060	-0.061	0.042	-0.070*	-0.025	-0.057	0.115***	0.054	-0.036	-0.005	-0.256***	0.010	0.275***	1	
LnFirmAge (20)	-0.135***	-0.136***	-0.158***	-0.120***	-0.183***	0.037	0.056	0.118***	0.006	0.286***	-0.095**	-0.269***	-0.054	0.281***	-0.007	-0.078*	-0.036	0.236***	0.050	1

*0.1, **0.05, ***0.01.

### 5.2. Main estimation results (testing H1)

The main results are presented in Table 4. The first two columns and the last two columns show OLS estimation results, while the middle two columns present Poisson regression results. In the OLS estimation, we used the logarithmic value of audit report lag (*LnLag*), whereas in the Poisson regression, we employed the raw value of audit report lag (*Lag*).

**Table 4:** Main results (testing H1).

	(1)	(2)	(3)	(4)	(5)	(6)
	OLS	OLS	Poisson	Poisson	OLS	OLS
VARIABLES	LnLag	LnLag	Lag	Lag	AdjLag	AdjLag
Constant	4.468***	4.478***	4.419***	4.430***	0.492***	0.503***
	(0.149)	(0.147)	(0.111)	(0.110)	(0.160)	(0.158)
LnTotalKAM	0.031**		0.033***		0.031*	
	(0.015)		(0.011)		(0.016)	
AdjKAM		0.013**		0.014***		0.014**
		(0.006)		(0.004)		(0.006)
AuditorEdu	-0.027	-0.030	-0.030**	-0.033**	-0.028	-0.032*
	(0.020)	(0.019)	(0.014)	(0.014)	(0.018)	(0.018)
AuditorGender	-0.037	-0.038	-0.022	-0.023	-0.036	-0.037
	(0.024)	(0.024)	(0.018)	(0.018)	(0.028)	(0.028)
AuditorAbroad	0.052***	0.050**	0.054***	0.052***	0.051**	0.049**
	(0.020)	(0.019)	(0.014)	(0.014)	(0.022)	(0.022)
LnAuditorTenure	0.004	0.007	0.005	0.008	0.001	0.004
	(0.013)	(0.013)	(0.009)	(0.009)	(0.013)	(0.013)
LnAFSize	-0.011*	-0.010*	-0.009*	-0.008*	-0.009*	-0.008*
	(0.006)	(0.006)	(0.004)	(0.004)	(0.005)	(0.005)
Duality	0.034	0.035*	0.030*	0.031**	0.037**	0.039**
	(0.022)	(0.021)	(0.015)	(0.015)	(0.018)	(0.018)
BoardFemale	0.078	0.066	0.063*	0.054	0.080*	0.068
	(0.052)	(0.051)	(0.037)	(0.037)	(0.046)	(0.045)
BoardInd	-0.293**	-0.289**	-0.209*	-0.205*	-0.265*	-0.259*
	(0.144)	(0.142)	(0.107)	(0.106)	(0.153)	(0.152)
LnBoardSize	-0.162***	-0.168***	-0.145***	-0.151***	-0.156***	-0.163***
	(0.040)	(0.040)	(0.030)	(0.030)	(0.048)	(0.048)
Leverage	-0.041	-0.037	-0.026	-0.022	-0.044	-0.038
	(0.039)	(0.037)	(0.028)	(0.027)	(0.035)	(0.034)
Loss	0.092***	0.086***	0.083***	0.077***	0.098***	0.091***
	(0.020)	(0.019)	(0.014)	(0.014)	(0.017)	(0.017)
MTB	-0.043	-0.041	-0.047*	-0.045*	-0.049***	-0.047***
	(0.031)	(0.031)	(0.025)	(0.025)	(0.014)	(0.015)
LnSize	0.002	0.002	0.001	0.001	0.002	0.002
	(0.006)	(0.006)	(0.004)	(0.004)	(0.006)	(0.005)
Growth	-0.015	-0.013	-0.010	-0.008	-0.014	-0.012
	(0.026)	(0.026)	(0.019)	(0.019)	(0.027)	(0.027)
LnAge	-0.049**	-0.042**	-0.046***	-0.040***	-0.050**	-0.043**
	(0.020)	(0.020)	(0.014)	(0.014)	(0.022)	(0.022)
Year and Industry Fixed	Yes	Yes	Yes	Yes	Yes	Yes
Observations	602	602	602	602	602	602
R^2^/Pseudo R^2^	0.214	0.207	0.051	0.049	0.164	0.157
F Value/LR chi^2^	5.43***	5.32***	241.66***	236.09***	4.29***	4.04***

*LnLag*: The logarithmic value of the total number of the days following the fiscal year-end until the date of the audit report, *Lag*: The total number of the days following the fiscal year-end until the date of the audit report, *AdjLag*: Company’s audit report lag (days) minus the mean value of audit report lag (days) for industry-year, *LnTotalKAM*: Logarithmic value of total number of KAMs, *AdjKAM*: Total number of KAMs in the audit report of company minus the mean number of KAMs for industry-year, *AuditorEdu*: 1 if individual auditor holds masters’ or Ph.D. degree in a related field, 0 otherwise, *AuditorGender*: 1 if individual auditor is female, 0 otherwise, *AuditorAbroad*: 1 if individual auditor has an abroad experience, 0 otherwise, *LnAuditorTenure*: Logarithmic value of the duration of company and individual auditor relationship, *LnAFSize*: Logarithmic value of total number of employees in the audit firm, *Duality*: 1 if CEO and Chairperson is the same person, 0 otherwise, *BoardInd*: The percentage of independent directors on the board of company, *BoardFemale*: The percentage of female members on the board of company: *LnBoardSize*: The logarithmic value of the total number of board members, *Leverage*: Total debts are divided by total assets, *Loss*: 1 if company reports loss, 0 otherwise, *MTB*: Market to book value, *LnSize*: The logarithmic value of total assets, Growth: The change in total sales of company, *LnAge*: The natural logarithmic value of years since the company formation.

* 0.1, **0.05, ***0.01, and standard errors are in parentheses.

The results of both the OLS and Poisson estimations show that the total number of KAMs (*LnTotalKAM*) and the difference between a company’s KAMs and the industry average (*AdjKAM*) increase audit report lag, whether measured in logarithmic (coefficients: 0.031 and 0.013), raw (0.033 and 0.014), or adjusted reporting lag values (0.031 and 0.014) of companies (LnLag, Lag, and AdjLag). Specifically, a one-unit increase in the logarithmic value of the KAM number causes an average increase of 0.03 in companies’ audit report lag, whereas a one-unit increase in the industry average KAM number causes an average increase of 0.01. This may be because individual auditors and audit firms exert more effort and take more time to report the KAMs in audit reports [8–10]. Accordingly, the number of KAMs in audit reports has a positive effect on audit report lag. Our findings indicate that individual auditors or audit firms invest more effort [8] and time preparing and reviewing audit reports to meet KAM requirements when performing procedures related to KAM items [11,12,52]. Consequently, as the number of KAMs reported increases, so does the delay in audit reporting. We accept H1.

For audit firm and individual auditor control variables, we find a negative association between audit firm size (*LnAFSize*) and audit report lag (*LnLag*, *Lag*, and *AdjLag*). Larger audit firms, with more resources and larger staff, can complete audit work more quickly than smaller firms [13,54]. Additionally, we find that individual auditors with international experience (*AuditorAbroad*) tend not to audit companies’ financial statements promptly (LnLag, Lag, and AdjLag), possibly because such auditors may lack awareness of local economic conditions, increasing the audit report lag [68].

Corporate governance variables at the company level indicate a positive, significant association between duality (Duality) and audit report lag. When the chairperson and CEO roles are combines (*Duality*), companies are more susceptible to financial statement irregularities, leading to longer reporting lags due to extended audit procedures [75]. Board independence (*BoardInd*) and board size (*LnBoardSize*) are negatively and significantly associated with audit report lag, as independent board members (*BoardInd*) are seen as overseeing company management [74], and larger boards (*LnBoardSize*) are more effective in their duties, leading to shorter reporting lags [46,74].

Audit report lag is higher for companies reporting a loss (*Loss*), as these companies may delay the disclosure of bad news (*Loss*) [71]. Older companies (*LnAge*) and those with higher market-to-book values (*MTB*) are more likely to be audited promptly (*LnLag*, *Lag*, and *AdjLag*), as older companies tend to have stronger internal control systems [69].

### 5.3. The results of interaction variables (testing H2)

We created interaction variables using audit firm size (*LnAFSize*) and the total number of KAMs (*LnTotalKAM* and *AdjKAM*) to test the moderating effect of audit firm size on audit report lag. We re-ran our base model, adding the interaction variables (LnTotalKAM*LnAFSize and AdjKAM*LnAFSize). Table 5 results for these interaction variables indicate that audit firm size moderates the effect of KAMs on audit report lag (*LnLag*, *Lag*, and *AdjLag*) (coefficients are -0.019, -0.009, -0.014, -0.007, -0.019, and -0.009).

**Table 5:** The results of interaction variables (Testing H2).

	(1)	(2)	(3)	(4)	(5)	(6)
	OLS	OLS	Poisson	Poisson	OLS	OLS
VARIABLES	LnLag	LnLag	Lag	Lag	AdjLag	AdjLag
Constant	4.379***	4.407***	4.354***	4.380***	0.404**	0.433***
	(0.154)	(0.150)	(0.115)	(0.112)	(0.158)	(0.154)
LnTotalKAM	0.116***		0.094***		0.115***	
	(0.042)		(0.031)		(0.040)	
AdjKAM		0.053***		0.043***		0.053***
		(0.018)		(0.013)		(0.016)
LnTotalKAM* LnAFSize	-0.019**		-0.014**		-0.019**	
	(0.009)		(0.006)		(0.009)	
AdjKAM * LnAFSize		-0.009**		-0.007**		-0.009**
		(0.004)		(0.003)		(0.004)
AuditorEdu	-0.027	-0.029	-0.030**	-0.033**	-0.028	-0.032*
	(0.020)	(0.019)	(0.014)	(0.014)	(0.018)	(0.018)
AuditorGender	-0.040	-0.041*	-0.024	-0.026	-0.038	-0.041
	(0.024)	(0.024)	(0.018)	(0.018)	(0.027)	(0.027)
AuditorAbroad	0.049**	0.046**	0.052***	0.049***	0.048**	0.046**
	(0.020)	(0.020)	(0.014)	(0.014)	(0.022)	(0.022)
LnAuditorTenure	0.004	0.006	0.005	0.007	0.001	0.003
	(0.013)	(0.013)	(0.009)	(0.009)	(0.014)	(0.013)
LnAFSize	-0.001	-0.007	-0.002	-0.006	-0.001	-0.005
	(0.008)	(0.006)	(0.005)	(0.004)	(0.007)	(0.006)
Duality	0.033	0.035	0.029*	0.030*	0.036*	0.038**
	(0.022)	(0.021)	(0.015)	(0.015)	(0.018)	(0.018)
BoardFemale	0.073	0.062	0.061	0.052	0.076*	0.064
	(0.052)	(0.051)	(0.037)	(0.037)	(0.046)	(0.045)
BoardInd	-0.300**	-0.310**	-0.215**	-0.221**	-0.271*	-0.279*
	(0.145)	(0.143)	(0.107)	(0.106)	(0.154)	(0.153)
LnBoardSize	-0.165***	-0.174***	-0.147***	-0.155***	-0.159***	-0.168***
	(0.040)	(0.040)	(0.030)	(0.030)	(0.048)	(0.048)
Leverage	-0.031	-0.030	-0.019	-0.017	-0.034	-0.031
	(0.039)	(0.038)	(0.029)	(0.028)	(0.036)	(0.035)
Loss	0.092***	0.088***	0.082***	0.078***	0.098***	0.093***
	(0.020)	(0.019)	(0.014)	(0.014)	(0.018)	(0.017)
MTB	-0.039	-0.035	-0.044*	-0.041	-0.045***	-0.041***
	(0.031)	(0.031)	(0.025)	(0.025)	(0.014)	(0.015)
LnSize	0.004	0.005	0.002	0.003	0.004	0.004
	(0.006)	(0.006)	(0.004)	(0.004)	(0.006)	(0.006)
Growth	-0.016	-0.013	-0.010	-0.007	-0.015	-0.012
	(0.026)	(0.026)	(0.019)	(0.019)	(0.027)	(0.027)
LnAge	-0.044**	-0.039*	-0.042***	-0.037**	-0.046**	-0.040*
	(0.020)	(0.020)	(0.015)	(0.014)	(0.022)	(0.022)
Year and Industry Fixed	Yes	Yes	Yes	Yes	Yes	Yes
Observations	602	602	602	602	602	602
R^2^/Pseudo R^2^	0.221	0.215	0.052	0.050	0.171	0.176
F Value/LR chi^2^	5.42***	5.36***	246.35***	241.65***	4.28***	4.18***

*LnLag*: The logarithmic value of the total number of the days following the fiscal year-end until the date of the audit report, *Lag*: The total number of the days following the fiscal year-end until the date of the audit report, *AdjLag*: Company’s audit report lag (days) minus the mean value of audit report lag (days) for industry-year, *LnTotalKAM*: Logarithmic value of total number of KAMs, *AdjKAM*: Total number of KAMs in the audit report of company minus the mean number of KAMs for industry-year, *AuditorEdu*: 1 if individual auditor holds masters’ or Ph.D. degree in a related field, 0 otherwise, *AuditorGender*: 1 if individual auditor is female, 0 otherwise, *AuditorAbroad*: 1 if individual auditor has an abroad experience, 0 otherwise, *LnAuditorTenure*: Logarithmic value of the duration of company and individual auditor relationship, *LnAFSize*: Logarithmic value of total number of employees in the audit firm, *Duality*: 1 if CEO and Chairperson is the same person, 0 otherwise, *BoardInd*: The percentage of independent directors on the board of company, *BoardFemale*: The percentage of female members on the board of company: *LnBoardSize*: The logarithmic value of the total number of board members, *Leverage*: Total debts are divided by total assets, *Loss*: 1 if company reports loss, 0 otherwise, *MTB*: Market to book value, *LnSize*: The logarithmic value of total assets, Growth: The change in total sales of company, *LnAge*: The natural logarithmic value of years since the company formation.

* 0.1, **0.05, ***0.01, and standard errors are in parentheses.

Large audit firms can issue audit reports in a timely manner, even when reporting many KAMs. This result is likely due to large audit firms having greater staff and resources and more advanced technology compared to smaller firms [15,54,56]. Thus, large audit firms can mitigate the effect of KAMs on audit report lag. Although the number of KAMs generally causes reporting lags, companies audited by large firms experience reduced audit report lag, even when KAM counts increase.

### 5.4. The impact of different types of KAMs on audit report lag (testing H3)

To examine how different types of KAMs affect audit report lag, we categorized KAMs based on their relation to key financial statements, such as the balance sheet and income statement. These categories are asset-related KAMs (*LnTotalKAMType*_*Assets*_ or *AdjKAMType*_*Assets*_), liability-related KAMs (*LnTotalKAMType*_*Liabilities*_ or *AdjKAMType*_*Liabilities*_), revenues-related KAMs (*LnTotalKAMType*_*Revenues*_ or *AdjKAMType*_*Revenues*_) and other-related KAMs (*LnTotalKAMType*_*Others*_ or *AdjKAMType*_*Others*_), where “other” refers to KAM disclosures not directly tied to the balance sheet or income statement. We reran our main estimation, including these KAM sub-categories.

Our results indicate that all categories positively affect audit report lag, but only revenues-related KAMs are significant (coefficients are 0.030, 0.032, 0.032, 0.029, 0.031, and 0.031). Table 6 presents these results, which align with Filipović et al. [57] and Pérez et al. [58], who emphasize the importance of revenue items in KAMs. Individual auditors spend considerable time on these items, as supported by Horvat et al. [16], and our findings also support these claims.

**Table 6:** The results regarding sub-categories of KAMs (Testing H3).

	(1)	(2)	(3)	(4)	(5)	(6)
VARIABLES	LnLag	Lag	AdjLag	LnLag	Lag	AdjLag
Constant	4.345***	4.294***	0.359**	4.443***	4.396***	0.462***
	(0.171)	(0.153)	(0.169)	(0.151)	(0.112)	(0.149)
LnTotalKAMType_Assets_	0.019	0.022	0.019			
	(0.017)	(0.016)	(0.017)			
LnTotalKAMType_Liabilities_	0.021	0.022	0.026			
	(0.024)	(0.024)	(0.024)			
LnTotalKAMType_Revenues_	0.030*	0.032*	0.032*			
	(0.018)	(0.018)	(0.017)			
LnTotalKAMType_Others_	0.022	0.020	0.024			
	(0.036)	(0.032)	(0.035)			
AdjKAMType_Assets_				0.020	0.023*	0.019
				(0.018)	(0.013)	(0.017)
AdjKAMType_Liabilities_				0.022	0.022	0.026
				(0.022)	(0.016)	(0.022)
AdjKAMType_Revenues_				0.029*	0.031**	0.031*
				(0.016)	(0.012)	(0.016)
AdjKAMType_Others_				0.020	0.019	0.026
				(0.040)	(0.029)	(0.039)
AuditorEdu	-0.030	-0.033*	-0.033*	-0.030	-0.033**	-0.033*
	(0.018)	(0.017)	(0.018)	(0.019)	(0.014)	(0.019)
AuditorGender	-0.038	-0.024	-0.038	-0.039	-0.024	-0.038
	(0.029)	(0.029)	(0.028)	(0.024)	(0.018)	(0.024)
AuditorAbroad	0.049**	0.052**	0.049**	0.050**	0.052***	0.049**
	(0.022)	(0.021)	(0.022)	(0.020)	(0.014)	(0.019)
LnAuditorTenure	0.007	0.008	0.004	0.007	0.007	0.003
	(0.014)	(0.013)	(0.013)	(0.013)	(0.009)	(0.013)
LnAFSize	-0.011*	-0.009*	-0.009	-0.011*	-0.009*	-0.009
	(0.005)	(0.005)	(0.005)	(0.006)	(0.004)	(0.006)
Duality	0.035*	0.031*	0.038**	0.035	0.031**	0.038*
	(0.018)	(0.018)	(0.018)	(0.021)	(0.015)	(0.021)
BoardFemale	0.077	0.065	0.081*	0.075	0.063*	0.080
	(0.047)	(0.043)	(0.047)	(0.052)	(0.037)	(0.051)
BoardInd	-0.274*	-0.191	-0.242	-0.274*	-0.190*	-0.241*
	(0.156)	(0.139)	(0.153)	(0.143)	(0.106)	(0.142)
LnBoardSize	-0.162***	-0.144***	-0.156***	-0.162***	-0.144***	-0.155***
	(0.049)	(0.044)	(0.048)	(0.041)	(0.030)	(0.040)
Leverage	-0.047	-0.032	-0.049	-0.046	-0.031	-0.048
	(0.035)	(0.034)	(0.035)	(0.038)	(0.028)	(0.038)
Loss	0.089***	0.081***	0.094***	0.089***	0.080***	0.094***
	(0.017)	(0.016)	(0.017)	(0.020)	(0.014)	(0.019)
MTB	-0.042**	-0.046***	-0.047***	-0.042	-0.046*	-0.047
	(0.017)	(0.017)	(0.015)	(0.031)	(0.025)	(0.031)
LnSize	0.004	0.003	0.003	0.004	0.002	0.003
	(0.006)	(0.005)	(0.005)	(0.006)	(0.004)	(0.006)
Growth	-0.014	-0.008	-0.013	-0.015	-0.010	-0.014
	(0.028)	(0.025)	(0.027)	(0.026)	(0.019)	(0.026)
LnAge	-0.043**	-0.040*	-0.044**	-0.043**	-0.040***	-0.044**
	(0.022)	(0.023)	(0.022)	(0.020)	(0.014)	(0.020)
Year and Sector Fixed	Yes	Yes	Yes	Yes	Yes	Yes
Observations	598	598	598	598	598	598
R^2^/Pseudo R^2^	0.209	0.049	0.159	0.209	0.049	0.160
F Value/LR chi^2^	4.95***	155.47***	3.69***	4.82***	237.68***	3.47***

*LnLag*: The logarithmic value of the total number of the days following the fiscal year-end until the date of the audit report, *Lag*: The total number of the days following the fiscal year-end until the date of the audit report, *AdjLag*: Company’s audit report lag (days) minus the mean value of audit report lag (days) for industry-year, *LnTotalKAMType*_*Assets*_:Logarithmic value of total number of KAMs regarding companies’ assets, *LnTotalKAMType*_*Liabilities*_: Logarithmic value of total number of KAMs regarding companies’ liabilities, LnTotalKAMType_Revenues_: Logarithmic value of total number of KAMs regarding companies’ revenues, LnTotalKAMType_Others_:Logarithmic value of total number of KAMs regarding other items, AdjKAMType_Assets_: Total number of KAMs regarding assets in the audit report of company minus the mean number of KAMs regarding assets for industry-year; AdjKAMType_Liabilities,_ Total number of KAMs regarding liabilities in the audit report of company minus the mean number of KAMs regarding liabilities for industry-year, AdjKAMType_Revenues_: Total number of KAMs regarding revenues in the audit report of company minus the mean number of KAMs regarding revenues for industry-year, AdjKAMType_Others_: Total number of KAMs regarding other items in the audit report of company minus the mean number of KAMs regarding other items for industry-year, *AuditorEdu*: 1 if individual auditor holds masters’ or Ph.D. degree in a related field, 0 otherwise, *AuditorGender*: 1 if individual auditor is female, 0 otherwise, *AuditorAbroad*: 1 if individual auditor has an abroad experience, 0 otherwise, *LnAuditorTenure*: Logarithmic value of the duration of company and individual auditor relationship, *LnAFSize*: Logarithmic value of total number of employees in the audit firm, *Duality*: 1 if CEO and Chairperson is the same person, 0 otherwise, *BoardInd*: The percentage of independent directors on the board of company, *BoardFemale*: The percentage of female members on the board of company: *LnBoardSize*: The logarithmic value of the total number of board members, *Leverage*: Total debts are divided by total assets, *Loss*: 1 if company reports loss, 0 otherwise, *MTB*: Market to book value, *LnSize*: The logarithmic value of total assets, Growth: The change in total sales of company, *LnAge*: The natural logarithmic value of years since the company formation.

*0.1, **0.05, ***0.01, and standard errors are in parentheses.

### 5.5. Robustness analysis: System GMM

To address endogeneity concerns, we use system GMM models for robustness checks. The reliability of GMM estimators relies on the absence of second-order autocorrelation in the error term and the validity of the instruments [77]. According to Li et al. [78] that system GMM is appropriate if the dataset spans at least three time periods and the independent variable is time-variant. Thus, the GMM estimation procedure is applied when fixed-effect regressions of the current values of the dependent variable are regressed on its lagged values, provided that the coefficients of these lagged values are statistically significant. These conditions are satisfied in the current analysis [78].

Table 7 presents system GMM results, with post estimation results (i.e., AR and Hansen tests) shown at the bottom. Results from the AR (1), AR (2), Hansen and diff-in Hansen tests confirm the absence of second-order correlation and validate the instruments used. The coefficients of LnTotalKAM and AdjKAM (0.014, 0.018, 3.781, 1.191, 0.043, and 0.018, respectively) align with the main estimation results when accounting for potential endogeneity. These findings confirm that endogeneity is not a significant issue in this analysis.

**Table 7:** System GMM results (robust results for H1).

	(1)	(2)	(3)	(4)	(5)	(6)
VARIABLES	LnLag	LnLag	Lag	Lag	AdjLag	AdjLag
Constant	4.547***	5.328***	-17.59	33.51	-1.210	-0.581
	(0.474)	(0.678)	(55.84)	(44.77)	(0.824)	(0.673)
L.LnLag	-0.484***	-0.460***				
	(0.009)	(0.030)				
L.Lag			-0.535***	-0.541***		
			(0.031)	(0.029)		
L.AdjLag					-0.530***	-0.502***
					(0.028)	(0.027)
LnTotalKAM	0.014**		3.781**		0.043**	
	(0.005)		(1.510)		(0.021)	
AdjKAM		0.018*		1.191*		0.018*
		(0.011)		(0.625)		(0.009)
AuditorEdu	-0.020***	-0.024	-0.700	-0.063	-0.010	-0.008
	(0.004)	(0.022)	(1.674)	(1.423)	(0.024)	(0.023)
AuditorGender	-0.005	-0.048**	-1.232	-3.049**	-0.012	-0.015
	(0.006)	(0.018)	(1.583)	(1.228)	(0.021)	(0.020)
AuditorAbroad	0.034***	0.002	-0.433	0.326	-0.005	-0.017
	(0.006)	(0.031)	(1.764)	(1.807)	(0.028)	(0.029)
LnAuditorTenure	0.029***	0.026**	1.816***	1.606***	0.030***	0.024**
	(0.002)	(0.010)	(0.638)	(0.607)	(0.010)	(0.010)
LnAFSize	-0.063***	-0.068***	-4.791***	-4.292***	-0.072***	-0.063***
	(0.004)	(0.013)	(0.787)	(0.831)	(0.014)	(0.015)
Duality	0.040	0.026	-4.163	-8.215	0.034	0.005
	(0.033)	(0.091)	(6.052)	(5.810)	(0.103)	(0.104)
BoardFemale	0.0092	0.115	6.048	2.149	0.0831	0.034
	(0.022)	(0.099)	(6.526)	(5.976)	(0.096)	(0.087)
BoardInd	-0.034	-0.480***	-40.35***	-44.71***	-0.316**	-0.302*
	(0.024)	(0.156)	(9.148)	(9.160)	(0.155)	(0.159)
LnBoardSize	-0.208***	-0.197***	-15.71***	-15.79***	-0.202***	-0.199***
	(0.019)	(0.059)	(3.713)	(3.710)	(0.061)	(0.055)
Leverage	-0.102***	0.084	10.16	8.066	-0.042	0.069
	(0.015)	(0.111)	(6.374)	(5.753)	(0.108)	(0.098)
Loss	-0.001	-0.005	0.829	0.358	0.016	-0.001
	(0.003)	(0.014)	(0.777)	(0.811)	(0.014)	(0.014)
MTB	0.053***	0.063*	3.228	4.201	0.031	0.021
	(0.004)	(0.035)	(2.815)	(2.722)	(0.033)	(0.026)
LnSize	-0.023***	-0.038*	-1.463	-1.300	-0.012	-0.014
	(0.008)	(0.021)	(1.441)	(1.411)	(0.024)	(0.023)
Growth	-0.056***	-0.039**	-2.923***	-2.350**	-0.040**	-0.037**
	(0.003)	(0.016)	(0.996)	(0.934)	(0.017)	(0.014)
LnAge	0.045	0.160*	14.66**	13.70**	0.096	0.078
	(0.047)	(0.089)	(5.859)	(5.763)	(0.097)	(0.090)
Year and Industry Fixed	Yes	Yes	Yes	Yes	Yes	Yes
Observations	453	453	453	453	453	453
AR (1) p value	0.02	0.03	0.03	0.05	0.03	0.05
AR (2) p value	0.62	0.63	0.36	0.45	0.35	0.41
Hansen p value	0.79	0.55	0.51	0.26	0.50	0.22
Diff-in Hansen p value	0.76	0.45	0.38	0.16	0.37	0.14

*L.LnLag*: The Lagged logarithmic value of the total number of the days following the fiscal year-end until the date of the audit report *LnLag*: The logarithmic value of the total number of the days following the fiscal year-end until the date of the audit report, *L.Lag*: The lagged value of total number of the days following the fiscal year-end until the date of the audit report, *Lag*: The total number of the days following the fiscal year-end until the date of the audit report, *L.AdjLag*: The lagged value of company’s audit report lag (days) minus the mean value of audit report lag (days) for industry-year *AdjLag*: Company’s audit report lag (days) minus the mean value of audit report lag (days) for industry-year, *LnTotalKAM*: Logarithmic value of total number of KAMs, *AdjKAM*: Total number of KAMs in the audit report of company minus the mean number of KAMs for industry-year, *AuditorEdu*: 1 if individual auditor holds masters’ or Ph.D. degree in a related field, 0 otherwise, *AuditorGender*: 1 if individual auditor is female, 0 otherwise, *AuditorAbroad*: 1 if individual auditor has an abroad experience, 0 otherwise, *LnAuditorTenure*: Logarithmic value of the duration of company and individual auditor relationship, *LnAFSize*: Logarithmic value of total number of employees in the audit firm, *Duality*: 1 if CEO and Chairperson is the same person, 0 otherwise, *BoardInd*: The percentage of independent directors on the board of company, *BoardFemale*: The percentage of female members on the board of company: *LnBoardSize*: The logarithmic value of the total number of board members, *Leverage*: Total debts are divided by total assets, *Loss*: 1 if company reports loss, 0 otherwise, *MTB*: Market to book value, *LnSize*: The logarithmic value of total assets, Growth: The change in total sales of company, *LnAge*: The natural logarithmic value of years since the company formation.

*0.1, **0.05, ***0.01, and standard errors are in parentheses.

### 5.6. Additional analysis: Determinants of KAMs

Recent studies have explored the determinants of KAMs in relation to various internal and external factors of companies [45,49,62]. The determinants of KAM disclosures may depend on company characteristics (e.g., complexity, size, life cycle), audit firm attributes (e.g., size), and individual auditor demographics (e.g., gender, experience) [79–82]. In an additional analysis in this paper, we examine KAMs based on individual auditor attributes, audit firm size, corporate governance, and company-specific characteristics, using data from an emerging market. The estimation model is as follows:

LnTotalKAM (or AdjKAM) =  β_0_ +  β_1_ AuditorEdu +  β_2_ AuditorGender +β_3_ AuditorAbroad +  β_4_ LnAuditorTenure +  β_5_ LnAFSize +  β_6_ Duality +  β_7_ BoardInd +  β_8_ BoardFemale +  β_9_ LnBoardSize +  β_10_ Leverage +  β_11_ Loss +  β_12_ MTB +  β_13_ LnSize +  β_14_ Growth +  β_15_ LnAge +  Industry Fixed +  Year Fixed +  error term.

In this analysis, we aim to identify the determinants of KAMs without positing specific hypotheses. We present and explain why these variables may influence the number of KAMs. Control variables from pervious sections are included to examine their relationship with audit report lag. Female auditors (*AuditorGender*), known for their risk aversion [83], may report more KAMs, reflecting a higher level of engagement. Additionally, an auditor’s educational background (*AuditorEdu*) impacts their perception and handling of audit complexities and risks, with those holding advanced degrees potentially reporting a greater number of KAMs [84]. Audit firm or individual auditor tenure (*LnAuditorTenure*) may also affect KAM reporting, as familiarity with the client can reduce the number of KAMs reported. Recent highlight the influence of auditor changes on KAM reporting [85].

Rahaman and Karim [49] and Elmarzouky et al. [43], suggest that board structure affects the number of KAMs. Female (*BoardFemale*) and independent directors (*BoardInd*) may seek detailed disclosure of KAMs to enhance reporting quality due to their risk-averse nature or their effective roles in monitoring and oversight. Larger boards or committees (*LnBoardSize*) provides better monitoring of corporate affairs and improved interactions with auditors, which may increase the amount of KAM reporting. According to Noureldeen et al. [86], CEO duality (*Duality*) is associated with weaker corporate governance and higher risks, prompting more extensive KAM disclosures by auditors as required by regulators.

Individual auditors report more KAMs for large companies (*LnSize*), older companies (*LnAge*) and high leveraged companies (*Leverage*) due to increased litigation risks and complexity [79,84,87]. Companies reporting losses (*Loss*) may face going concern problems, prompting auditors to disclose more KAMs [79]. Similarly, higher growth rates (*Growth*) suggest elevated risks, encouraging auditors to report additional KAMs [84]. Additionally, company value (*MTB*) may influence the number of KAMs disclosed [85].

The results of the determinants of KAMs are summarized in Table 8. The first two columns present OLS estimation results, using the natural logarithmic value of the number of KAMs (*LnTotalKAM*) and industry-year adjusted KAMs (*AdjKAM*) as dependent variables. The last column provides Poisson regression results, using the raw count of total KAMs (*KAM*) as the dependent variable.

**Table 8:** Additional analysis: Determinants of KAMs.

	(1)	(2)	(3)
	OLS	OLS	Poisson
VARIABLES	LnTotalKAM	AdjKAM	TotalKAM
Constant	0.754*	-0.146	0.769
	(0.407)	(0.922)	(0.564)
AuditorEdu	0.148***	0.404***	0.198***
	(0.054)	(0.122)	(0.071)
AuditorGender	0.0596	0.237	0.087
	(0.067)	(0.152)	(0.090)
AuditorAbroad	-0.122**	-0.236*	-0.130
	(0.054)	(0.124)	(0.081)
LnAuditorTenure	-0.024	-0.043	-0.032
	(0.036)	(0.081)	(0.051)
LnAFSize	-0.114***	-0.255***	-0.121***
	(0.017)	(0.040)	(0.025)
Duality	-0.224***	-0.542***	-0.259***
	(0.059)	(0.134)	(0.086)
BoardFemale	0.124	0.351	0.197
	(0.142)	(0.320)	(0.200)
BoardInd	-0.795**	-1.525*	-0.646
	(0.394)	(0.889)	(0.566)
LnBoardSize	-0.317***	-0.539**	-0.212
	(0.111)	(0.252)	(0.158)
Leverage	0.005	-0.212	-0.069
	(0.107)	(0.237)	(0.150)
Loss	0.055	0.0253	0.008
	(0.055)	(0.124)	(0.076)
MTB	0.145*	0.303	0.120
	(0.086)	(0.196)	(0.097)
LnSize	0.105***	0.241***	0.114***
	(0.016)	(0.038)	(0.023)
Growth	0.115	0.276*	0.067
	(0.0732)	(0.164)	(0.094)
LnAge	-0.0591	-0.292**	-0.136*
	(0.056)	(0.126)	(0.078)
Year and Industry Fixed	Yes	Yes	Yes
Observations	602	602	602
R^2^/Pseudo R^2^	0.231	0.215	0.056
F Value/LR chi^2^	6.33***	5.89***	111.34***

*LnTotalKAM*: Logarithmic value of total number of KAMs, *AdjKAM*: Total number of KAMs in the audit report of company minus the mean number of KAMs for industry-year, *TotalKAM*: Total number of KAMs, *AuditorEdu*: 1 if individual auditor holds masters’ or Ph.D. degree in a related field, 0 otherwise, *AuditorGender*: 1 if individual auditor is female, 0 otherwise, *AuditorAbroad*: 1 if individual auditor has an abroad experience, 0 otherwise, *LnAuditorTenure*: Logarithmic value of the duration of company and individual auditor relationship, *LnAFSize*: Logarithmic value of total number of employees in the audit firm, *Duality*: 1 if CEO and Chairperson is the same person, 0 otherwise, *BoardInd*: The percentage of independent directors on the board of company, *BoardFemale*: The percentage of female members on the board of company: *LnBoardSize*: The logarithmic value of the total number of board members, *Leverage*: Total debts are divided by total assets, *Loss*: 1 if company reports loss, 0 otherwise, *MTB*: Market to book value, *LnSize*: The logarithmic value of total assets, Growth: The change in total sales of company, *LnAge*: The natural logarithmic value of years since the company formation.

*0.1, **0.05, ***0.01, and standard errors are in parentheses.

Individual auditors with master’s or Ph.D. degrees (*AuditorEdu*) are more likely to disclose a higher number of KAMs in audit reports. Conversely, larger audit firms (*LnAFSize*) tend to report fewer KAMs. Individual auditors with international experience (*AuditorAbroad*) also disclose fewer KAMs. Companies with CEO duality (*Duality*), larger boards (*LnBoardSize*), and a higher proportion of independent directors (*BoardInd*) are associated with fewer KAMs in their audit reports. However, larger firms (*LnSize*) generally disclose more KAMs, whereas older firms (*LnAge*) disclose fewer.

## 6. Conclusion

In our paper, we investigate the effect of the number of KAMs on audit report lag and examine how audit firm size moderates this relationship. We also explore the specific types of KAMs that affect audit report lag. Our analyses primarily employ OLS and Poisson regressions, supplemented by system GMM for robustness. Finally, we examine the determinants of KAMs across company, audit firm, individual auditor, and corporate governance levels.

Our key findings indicate that an increase in the number of KAMs is associated with a longer audit report lag. Additionally, audit firm size moderates the relationship between KAMs and audit report lag, with revenue-related KAMs exerting the most significant effect. The system GMM results further validate our main estimations. Various factors at the company, audit firm, individual auditor, and corporate governance levels also significantly influence the number of KAMs reported.

Based on our findings, several managerial and policy implications emerge. Companies and audit firms should allocate adequate time and resources early in the audit cycle to address complex audit areas, as an increased number of KAMs can lengthen audit report lag. Since larger audit firms may handle KAMs more efficiently, companies might consider engaging these firms for audits involving numerous or complex KAMs to ensure timely financial reporting.

Understanding which types of KAMs impact audit report lag enables management to prioritize and proactively address these areas by improving internal controls and documentation. Investment in advanced audit technologies can further streamline the audit process for complex KAMs, helping to reduce audit report lag.

Providing continuous training for internal audit teams on emerging risks and complex accounting areas can better prepare them to support external auditors. Strengthening collaboration between management and the audit team, through regular communication and early identification of potential KAMs, can lead to a more efficient audit process. Regularly reviewing and updating internal policies and procedures to address common KAMs and audit findings can help mitigate issues contributing to audit report lag. Implementing these policies can improve audit efficiency, reduce audit report lag, and ensure timely and accurate financial reporting.

Our paper has several limitations. First, our sample is limited to an emerging market, resulting in a smaller dataset compared to cross-country studies, which may limit the generalizability of our findings. Second, we did not control for certain individual auditor characteristics, such as overall audit experience and workload, which could influence KAM reporting. Third, following Bepari and Mollik [24], we examined the net change in the number of KAMs, but found no significant association with audit report lag. Additionally, while our study provides insights into the impact of KAMs on audit report lag in Turkey, the relevance of this information to non-professional investors may be limited.

Our use of system GMM primarily strengthens our main results; future studies could explore this methodology for additional research questions.

Future research could investigate the impact of KAMs on audit opinions, as KAMs may signal potential issues affecting future audit opinions. Stakeholders might also consider current KAMs in their decision-making, underscoring the practical importance of KAM disclosures. Using system GMM in future studies could enhance understanding of issues beyond those explored in the present research.

## Supporting information

S1 Table Variable definition and references.
(DOCX)
