# Supplementary material for: Further evidence regarding the effect of KAMs on audit report lag
Source: PLoS One. 2025 Mar 25;20(3):e0320183. doi: 10.1371/journal.pone.0320183 (PMC11936242; doi:10.1371/journal.pone.0320183)
Supplement: S1 Table — Variable definition and references. (DOCX) [file pone.0320183.s001.docx]

Appendix A. **Variable definition and references**

| Variable | Definition | Source |
| --- | --- | --- |
| LnLag | the logarithmic value of the total number of days from the fiscal year-end to the date of the audit report | Public disclose platform of Turkey |
| Lag | the total number of days from the fiscal year-end to the date of the audit report | Public disclose platform of Turkey |
| AdjLag | a company’s audit report lag (in days) minus the mean audit report lag (in days) for the industry-year. | Public disclose platform of Turkey |
| LnTotalKAM | the logarithmic value of the total number of KAMs | Audit report of companies |
| AdjKAM | the total number of KAMs in the company’s audit report minus the mean number of KAMs for the industry-year | Audit report of companies |
| LnTotalKAMType_Assets_ | the logarithmic value of the total number of KAMs related to a company’s assets. | Audit report of companies |
| LnTotalKAMType_Liabilities_ | the logarithmic value of the total number of KAMs related to a company’s liabilities. | Audit report of companies |
| LnTotalKAMType_Revenues_ | the logarithmic value of the total number of KAMs related to a company’s revenues | Audit report of companies |
| LnTotalKAMType_Others_ | the logarithmic value of the total number of KAMs related to other items | Audit report of companies |
| AdjKAMType_Assets_ | the total number of KAMs related to assets in the company’s audit report minus the mean number of KAMs related to assets for the industry-year | Audit report of companies |
| AdjKAMType_Liabilities_ | the total number of KAMs related to liabilities in the company’s audit report minus the mean number of KAMs related to liabilities for the industry-year | Audit report of companies |
| AdjKAMType_Revenues_ | the total number of KAMs related to revenues in the company’s audit report minus the mean number of KAMs related to revenues for the industry-year | Audit report of companies |
| AdjKAMType_Others_ | the total number of KAMs related to other items in the company’s audit report minus the mean number of KAMs related to other items for the industry-year | Audit report of companies |
| AuditorEdu | 1 if the individual auditor holds a masters’ or Ph.D. degree in a related field, 0 otherwise | Audit report of companies, LinkedIn |
| AuditorGender | 1 if the individual auditor is female, 0 otherwise | Audit report of companies, LinkedIn |
| AuditorAbroad | 1 if the individual auditor has international experience, 0 otherwise | Audit report of companies, LinkedIn |
| LnAuditorTenure | the logarithmic value of the duration of the company and individual auditor relationship | Audit report of companies |
| LnAFSize | the logarithmic value of the total number of employees in the audit firm. | Transparency reports of audit firms |
| Duality | 1 if the CEO and Chairperson are the same person, 0 otherwise | Activity reports of companies |
| BoardInd | the percentage of independent directors on the company’s board | Activity reports of companies |
| BoardFemale | the percentage of female members on the company’s board | Activity reports of companies |
| LnBoardSize | the logarithmic value of the total number of board members | Activity reports of companies |
| Leverage | total debts divided by total assets | Finnet |
| Loss | 1 if the company reports loss, 0 otherwise | Finnet |
| MTB | Market-to-book value | Finnet |
| LnSize | the logarithmic value of total assets | Finnet |
| Growth | the change in the total sales of the company | Finnet |
| LnAge | the natural logarithmic value of years since the company’s formation | Finnet |
| Sources: Manually collected by authors from activity reports, audit reports, transparency reports, LinkedIn. Company-specific characteristics obtained from Finnet database. | | |
